# A global score for skin environmental exposure: integrating UV radiation, heat, and pollution via fuzzy analytic hierarchy process

**DOI:** 10.1007/s00484-026-03145-0

**Published:** 2026-04-13

**Authors:** Marcelo de Paula Corrêa, Ivana Riera Pereira Bastos, Ana Letícia Campos Yamamoto

**Affiliations:** Instituto de Recursos Naturais, Universidade Federal de Itajubá, Av. BPS 1303, Itajubá, Minas Gerais 37500903 Brazil

**Keywords:** environmental dermatology, skin exposome, photodamage, oxidative stress, preventive dermatology, climate skin health

## Abstract

Environmental stressors such as ultraviolet radiation (UVR), air pollutants, and thermal stress interact synergistically to accelerate skin damage, yet most available indicators still assess these factors in isolation. The Skin Environmental Exposure Score (SEES) is a new and innovative fuzzy logic-based tool that integrates UVR, heat, ozone, and particulate matter into a single 0-1000 score, prioritizing UVR while quantitatively incorporating co‑exposures relevant to cutaneous health. The term “score” is deliberately adopted to emphasize a standardized, easily interpretable numerical outcome that facilitates communication with both scientific and non‑specialist audiences. Using local and global environmental datasets, SEES reveals that urban pollution markedly amplifies UVR-related risks and that heat stress can extend high-risk exposure windows beyond traditional midday peaks, including early morning and late afternoon periods. Distinct regional patterns emerge, with tropical areas experiencing persistent combined UVR-heat stress, while temperate regions are more strongly affected by pollution-driven risks. Based on these patterns, SEES supports evidence-based thresholds for sun-safety policies, including pollution-adjusted UV alerts, heat-adaptive photoprotection guidance, and identification of high-risk regions. For clinicians, SEES provides a practical, quantitative measure of the external skin exposome, directly linking climatic and pollution conditions to cutaneous risk and offering a more realistic basis for prevention counseling than UV or air-quality indices alone. This tool can inform skin cancer prevention, photoaging strategies, and the protection of vulnerable populations in routine clinical practice and public health campaigns. Future applications should extend SEES to long‑term climate scenarios to anticipate evolving patterns of environmental risk to the skin.

**Supplementary Information:**

The online version contains supplementary material available at 10.1007/s00484-026-03145-0.

## Introduction

The cumulative impact of daily environmental exposures poses significant threats to cutaneous and hair health, necessitating urgent attention to mitigate their deleterious effects. Solar ultraviolet radiation (UVR), atmospheric pollutants, and fluctuations in ambient temperature (T) and relative humidity (RH) act synergistically to compromise epidermal integrity and follicular function. Among these stressors, UVR represents the most well-established hazard, having been classified as a Group 1 human carcinogen by the International Agency for Research on Cancer (IARC, 1992). UVR exposure triggers significant adverse effects on several biological structures. Its dermatopathological consequences include photoaging, photodermatoses, and structural degradation of hair fibers through protein denaturation, pigmentation loss, and increased fragility (Lee 2009; De Vecchi et al. 2019). Additionally, UVR poses a well-established risk for ocular damage, contributing to conditions such as cataracts, pterygium, and photokeratitis (Löfgren, 2017; Neale et al. 2023).

The cutaneous effects of airborne pollutants are equally concerning, as the IARC has similarly recognized for their mutagenic and carcinogenic potential (IARC 2015). Particulate matter (PM), ozone (O₃), sulfur dioxide (SO₂), nitrogen oxides (NOₓ), carbon compounds (CO, CO₂), volatile organic compounds (VOCs), and polycyclic aromatic hydrocarbons (PAHs) have been implicated in diverse dermatological pathologies including accelerated aging, dyspigmentation, acneiform eruptions, atopic dermatitis, and neoplastic transformation (Puri et al. 2017; Roberts 2021). Surface-level O₃ induces particularly deleterious effects through oxidative stress mechanisms that overwhelm cutaneous antioxidant defenses, resulting in inflammatory cascades that manifest clinically as fine rhytides and wrinkles, impaired barrier function, and exacerbation of existing dermatoses (Mancebo and Wang 2015; Parrado et al. 2019). This oxidative damage is potentiated when O₃ exposure coincides with UVR, creating a proinflammatory milieu that amplifies photodamage (Ferrara et al. 2021). Additional pollutants, including SO₂ and NO₂, similarly contribute to oxidative injury, accelerating wrinkle formation and lentigines while compromising hair shaft integrity (Li et al. 2017; Liang et al. 2020; Appenzeller and Tsatsakids, 2012).

While the detrimental effects of air pollution on skin integrity and the pathogenesis of dermatological conditions are well documented, a growing body of evidence underscores its significant, parallel impact on ocular surface health. Epidemiological and clinical studies consistently link exposure to ambient air pollutants, particularly PM_2.5_ and PM_10_, NO₂, and VOCs, to a higher prevalence and severity of various ocular diseases. The primary conditions associated include dry eye disease (DED), in which pollutants disrupt the tear film and exacerbate inflammation; allergic conjunctivitis, driven by pollutant-augmented allergenicity and immune response; and ocular surface inflammatory disorders. Furthermore, chronic exposure to specific pollutants has been associated with more severe endpoints, such as glaucoma and age-related macular degeneration (AMD), suggesting systemic and neurovascular pathways of damage. Therefore, ocular morbidity represents a critical, though often underappreciated, public health consequence of air pollution, necessitating integrated environmental and clinical strategies to mitigate risk (Millen et al. 2024).

The interplay between T and RH further modulates cutaneous responses by influencing thermoregulation and barrier homeostasis. While empirical and computational models have characterized these relationships (Zhao et al. 2021), the clinical implications are profound. Xerotic conditions arising from elevated T and reduced RH contrast with the sebaceous activation and follicular occlusion promoted by humid environments, creating divergent pathways for irritant dermatitis, acne exacerbation, and dyshidrotic conditions (Xu et al. 2008). These microclimatic factors also influence photoprotective practices, where thermal discomfort from occlusive sunscreens may compromise thermoregulation and adherence to use (Garzón-Mosquera and Aragón-Vargas 2024). Thermal discomfort can also compromise hair growth and fiber texture (Monselise et al. 2017).

T and RH are also critically related to ocular health, acting as key environmental factors that exacerbate a wide range of pathologies. Elevated T function acts as a direct inflammatory stressor on the ocular surface, increasing susceptibility to infections such as viral conjunctivitis and ocular herpes. Furthermore, each degree Celsius of T rise can amplify the damaging effects of UVR by approximately 2%, intensifying the risks for cataracts and uveal melanoma. Shifts in humidity patterns and increased torrential rains alter the dispersal of allergens and create environments conducive to disease vectors, aggravating conditions like allergic conjunctivitis and infectious uveitis. The interaction of heat with atmospheric pollutants, such as tropospheric O_3_, further compounds the risk for dry eye disease and other inflammatory disorders (Echevarria-Lucas et al. 2017).

Current environmental health risk assessment tools, including the Ultraviolet Index (UVI) (WHO 2002), Air Quality Index (AQI) (WHO 2006), and thermal comfort metrics like the Humidex (Masterton and Richardson 1979), provide valuable but isolated perspectives on these exposures (Zhao et al. 2021). On the other hand, the external exposome framework, encompassing exposures to different external environmental factors (Wild 2012; Cecchi et al. 2018), demands an integrated approach, particularly for cutaneous pathophysiology, where synergistic interactions between UVR, pollution, and microclimate are well-established (Krutmann et al. 2017; Passeron et al. 2021). Given this complexity, recent studies have sought to integrate data to assess the external exposome by implementing environmental data management systems (Tagliaferro et al. 2024).

Recognizing the critical need for integrated tools to assess combined environmental risks to skin health, this study introduces the Skin Environmental Exposure Score (SEES), a novel metric employing the Fuzzy Analytical Hierarchy Process (F-AHP) methodology (Van Laarhoven and Pedrycz 1983) to quantify synergistic environmental risks to skin health. Designed to advance the characterization of the external exposome, the SEES provides a standardized, dimensionless scale that captures the complex interplay among UVR, atmospheric pollutants, and thermal factors, offering a unified approach to risk assessment that traditional indices fall short.

Although specifically designed for skin health, the SEES can also serve as a proper environmental parameter for preventing other external exposome‑related conditions, such as ocular diseases; however, because evidence on pollution and thermal stress effects on the eye is still relatively limited (Millen et al. 2024), the term SEES was retained to reflect its primary cutaneous focus.

To maximize clarity and utility, we deliberately adopted the term “score” rather than “index”, underscoring its role as a scaled quantitative measure rather than a composite of arbitrary units. Derived from a robust multi-criteria model that integrates climatic, pollution, and UVR variables, the SEES is converted to an intuitive 0-1000 scale. This transformation facilitates cross-location and temporal comparisons and enhances communication with scientific and public audiences. The acronym SEES was strategically chosen for its phonetic resemblance to the verb “to see”, reinforcing its purpose: to “make visible” the environmental risks affecting skin health. This dual meaning can be leveraged in outreach through resonant messaging, such as “SEES the environment, protects your skin” or “SEES what your skin faces daily”, bridging technical precision with public engagement.

## Methods and development

This study proposes a novel methodological framework to assess the combined effects of key environmental risk factors on skin health: ultraviolet radiation (UVR), heat stress, and air pollutants (O₃, PM, and other gases [OG] including SO₂, NO₂, VOC, and PAH). Each factor was assigned a risk coefficient (K_i_) based on established risk scales, and the results were integrated using a multicriteria approach that combined the Fuzzy Analytic Hierarchy Process (F-AHP) for weighted prioritization and sensitivity-optimized aggregation functions. The F-AHP addresses uncertainty in expert judgments using triangular fuzzy numbers, while comparative analysis of arithmetic and geometric means ensures robust risk quantification.

The methodology systematically evaluates synergistic interactions between meteorological and pollution variables, with rigorous validation through consistency checks (CR < 10%) and extreme-value sensitivity testing. Subsequent subsections detail the mathematical formulations, including hierarchical structuring, fuzzy-weight derivation, normalization procedures, and the construction of the final composite score. This approach advances conventional univariate assessments by providing a scalable, uncertainty-aware model for region-specific profiling of external exposome risk.

All F-AHP calculations, SEES computations, sensitivity analyses, and graphical outputs were performed using open‑source Python libraries, including NumPy, Pandas, scikit‑learn, and Seaborn, ensuring full reproducibility of the analytical workflow.

### The fuzzy analytical hierarchy process (F-AHP) to determine the weight of each factor

Fuzzy Analytic Hierarchy Process (F-AHP) is an advanced decision-making method that integrates fuzzy set theory with the Analytic Hierarchy Process (AHP; Saaty 1987) to handle uncertainty and imprecision in subjective judgments. The AHP is a multicriteria decision-making technique that assesses how a response variable (in our study, the environmental exposure risk) relates to a set of environmental predictors. However, subjective pairwise comparisons are prone to vagueness-type uncertainty, necessitating the use of fuzzy-based techniques (Buckley 1985; Chang 1996). The F-AHP method is an effective tool for assessing the influence of multiple factors on a given phenomenon, accounting for the complexity of the attributes involved (Liu et al. 2020). This approach enables the determination of the relative importance of each criterion (UVR, pollutants, and heat stress) through pairwise comparisons in a nonparametric analysis.

The robustness of F-AHP is further demonstrated by its widespread application in multicriteria decision-making for public health studies (Chao et al. 2008; Kim and Jung 2023). The F-AHP has been employed in diverse applications, including developing air quality health indexes (Gorai et al. 2015), assessing environmental and health risks and impacts (Zheng et al. 2012; Akash et al. 2024; Nguyen et al. 2025), water Management planning (Srdjevic and Medeiros 2008), disaster preparedness in clinical laboratories (Ortiz-Barrios et al.  2025), and identifying suitable locations for urban infrastructure (Caprioli and Bottero 2021).

The F-AHP method ensures the SEES’s reliability by determining each criterion’s relative importance through pairwise comparisons, referred to as Pairwise Hierarchic Levels (ϕ), based on their relative significance. With its adaptive nature, the method uses comparison consistency to ensure process reliability, adjusting values as needed. The F-AHP consists of four key steps: (i) representation of the relative importance through pairwise comparison, (ii) aggregation of fuzzy sets for group decisions and weights/priorities determination, (iii) defuzzification of fuzzy sets into crisp values for final comparison, and (iv) consistency measurement of judgments (Liu et al. 2020). This structured approach enables clear prioritization among different factors and ensures consistency assessment, yielding a robust and reliable index.

**Fig. 1:**
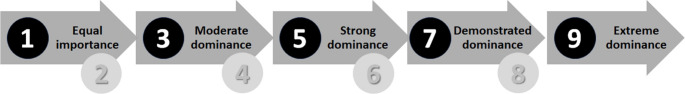
Pairwise hierarchic levels (φ) for comparison matrices in AHP

The decomposition phase involves constructing a hierarchy with the problem’s objective at the top level and criteria arranged in descending order. Subsequently, priorities must be established for elements relative to others within the hierarchical decision system. A pairwise comparison matrix is then constructed to determine comparative hierarchical levels using both the Saaty scale (Fig. 1) and fuzzy numbers (Table 1). The dominance relationship between ϕ values ranges from 1 to 9, where:

**Table 1:** Saaty’s pairwise comparisons and fuzzy triangular scales (Li et al. 2009)

Saaty Scale	Fuzzy Triangular Scale
1	(1,1,1)
2	(1,2,3)
3	(2,3,4)
4	(3,4,5)
5	(4,5,6)
6	(5,6,7)
7	(6,7,8)
8	(7,8,9)
9	(9,9,9)

- Level 1 indicates an equal contribution from both attributes toward the goal.
- Levels 3 and 5 represent a slight or strong preference for one attribute over another, respectively.
- Level 7 demonstrates the practical dominance of one attribute.
- The maximum level (9) indicates absolute evidence in favor of one attribute.
- Intermediate values (φ = 2, 4, 6, and 8) are used when finer gradations or compromises are required.

First, construct the fuzzy pairwise comparison matrix ($${\Phi}$$) as shown in Eq. (1). The relative weights of these hierarchical levels are calculated using fuzzy weight determination and defuzzification by computing the area centroid to obtain absolute weight values ($$\overrightarrow{v}$$). In the comparison matrix (Φ), each factor is compared pairwise with the others, with values assigned according to the φ level and expressed as triangular fuzzy numbers, according to $${}_{ij}=({l}_{ij}$$, $${m}_{ij},{u}_{ij})$$, using the numerical values ​​and relationships presented in Table 1. To avoid confusion with conventional matrix element notation (where *i* and *j* represent row and column indices), we denote each factor in the Φ matrix using Roman numerals.

1 $$\begin{array}{c}{\Phi}=\\\left(\begin{array}{ccc}(1,1,1)&({l}_{I,II},{m}_{I,II},{u}_{I,II})&\begin{array}{ccc}({l}_{I,III},{m}_{I,III},{u}_{I,III})&\cdots&({l}_{I,n},{m}_{I,n},{u}_{I,n})\end{array}\\(\raisebox{1ex}{$1$}\!\left/\!\raisebox{-1ex}{${\mathrm{u}}_{I,II}$}\right.,\raisebox{1ex}{$1$}\!\left/\!\raisebox{-1ex}{${m}_{I,II}$}\right.,\raisebox{1ex}{$1$}\!\left/\!\raisebox{-1ex}{${\mathrm{l}}_{I,II}$}\right.)&(1,1,1)&\begin{array}{ccc}({l}_{II,III},{m}_{II,III},{u}_{II,III})&\dots&({l}_{II,n},{m}_{II,n},{u}_{II,n})\end{array}\\\begin{array}{c}(\raisebox{1ex}{$1$}\!\left/\!\raisebox{-1ex}{${\mathrm{u}}_{I,III}$}\right.,\raisebox{1ex}{$1$}\!\left/\!\raisebox{-1ex}{${m}_{I,III}$}\right.,\raisebox{1ex}{$1$}\!\left/\!\raisebox{-1ex}{${\mathrm{l}}_{I,III}$}\right.)\\⋮\\(\raisebox{1ex}{$1$}\!\left/\!\raisebox{-1ex}{${\mathrm{u}}_{I,n}$}\right.,\raisebox{1ex}{$1$}\!\left/\!\raisebox{-1ex}{${m}_{I,n}$}\right.,\raisebox{1ex}{$1$}\!\left/\!\raisebox{-1ex}{${\mathrm{l}}_{}$}\right.)\end{array}&\begin{array}{c}(\raisebox{1ex}{$1$}\!\left/\!\raisebox{-1ex}{${\mathrm{u}}_{I,III}$}\right.,\raisebox{1ex}{$1$}\!\left/\!\raisebox{-1ex}{${m}_{I,III}$}\right.,\raisebox{1ex}{$1$}\!\left/\!\raisebox{-1ex}{${\mathrm{l}}_{I,III}$}\right.)\\⋮\\(\raisebox{1ex}{$1$}\!\left/\!\raisebox{-1ex}{${\mathrm{u}}_{n,II}$}\right.,\raisebox{1ex}{$1$}\!\left/\!\raisebox{-1ex}{${m}_{n,II}$}\right.,\raisebox{1ex}{$1$}\!\left/\!\raisebox{-1ex}{${\mathrm{l}}_{n,II}$}\right.)\end{array}&\begin{array}{ccc}(1,1,1)&\dots&({l}_{III,n},{m}_{III,n},{u}_{III,n})\\⋮&\ddots&\\(\raisebox{1ex}{$1$}\!\left/\!\raisebox{-1ex}{${\mathrm{u}}_{n,III}$}\right.,\raisebox{1ex}{$1$}\!\left/\!\raisebox{-1ex}{${m}_{n,III}$}\right.,\raisebox{1ex}{$1$}\!\left/\!\raisebox{-1ex}{${\mathrm{l}}_{n,III}$}\right.)&&(1,1,1)\end{array}\end{array}\right)\end{array}$$

Following Buckley’s (1985) methodology, we first compute the geometric mean (${\stackrel{\sim}{r}}_{i}$) using Eq. (2) and then determine the weights through Eq. (3). Consider the triangular fuzzy number ${}_{I,n}$= $({l}_{I,n},{m}_{I,n},{u}_{I,n})$ with the following operational laws:

2 $${\stackrel{\sim}{r}}_{i}={({\stackrel{\sim}{}}_{i1}\mathrm{x}\dots\mathrm{x}{\stackrel{\sim}{}}_{ij}\mathrm{x}\dots\mathrm{x}{\stackrel{\sim}{}}_{in})}^{1/n}$$

3 $${{\stackrel{\sim}{w}}_{i}={\stackrel{\sim}{r}}_{i}\times({\stackrel{\sim}{r}}_{1}+\cdots+{\stackrel{\sim}{r}}_{i}+\cdots+{\stackrel{\sim}{r}}_{n})}^{-1}$$

Here $\stackrel{\sim}{}$_𝑖𝑗_ represents the aggregated comparison value of criterion *i* relative to criterion *j*, ${\stackrel{\sim}{r}}_{i}$ is the geometric mean of the fuzzy comparisons between criterion *i* and all other criteria, and ${\stackrel{\sim}{w}}_{i}$ denotes the fuzzy weight of the *i*-th criterion, expressed as a triangular fuzzy number, ${\stackrel{\sim}{w}}_{i}$ = (𝑙𝑤_𝑖_, 𝑚𝑤_𝑖_, 𝑢𝑤_𝑖_), where 𝑙𝑤_𝑖_, 𝑚𝑤_𝑖_, 𝑢𝑤_𝑖_ correspond to the lower, middle, and upper values of the fuzzy weight, respectively.

The fuzzy weights are then converted to crisp values using Buckley’s (1985) centroid method for triangular fuzzy numbers:

4 $$C\left({\stackrel{\sim}{w}}_{i}\right)=\frac{{lw}_{i}+{mw}_{i}+{uw}_{i}}{3}$$

Subsequently, we normalize the weights using:

5 $${v}_{i}=\frac{C\left({\stackrel{\sim}{w}}_{i}\right)}{\sum_{i=1}^{n}C\left({\stackrel{\sim}{w}}_{i}\right)}$$

The priority vector (eigenvector $\overrightarrow{v}$), comprising components ${v}_{i}$, precisely quantifies each factor’s relative contribution. Note that the sum of all ${v}_{i}$values always equals 1.

6 $$\overrightarrow{v}=\left(\begin{array}{c}{v}_{1}\\{v}_{2}\\\begin{array}{c}{v}_{3}\\⋮\\{v}_{n}\end{array}\end{array}\right)$$

Consistency validation is critical and depends on the researcher’s weight assignments (Saaty and Vargas 2013). We evaluate this through:

7 $$\mathrm{C}\mathrm{I}=\frac{({{\uplambda}}_{\mathrm{m}\mathrm{a}\mathrm{x}}-\mathrm{n})}{(\mathrm{n}-1)}$$

8 $$\mathrm{C}\mathrm{R}=\frac{\mathrm{C}\mathrm{I}}{{\mathrm{R}\mathrm{I}}_{d}}$$

Where n is the order of the matrix, and λ_max_ is the principal eigenvalue of the comparison matrix Φ, calculated as:

9 $${\lambda}_{max}=\sum_{j=1}^{n}\frac{{\stackrel{\sim}{V}}_{i}}{n{\stackrel{\sim}{V}}_{i}}$$

The Random Consistency Index (${\mathrm{R}\mathrm{I}}_{d}$), proposed by Saaty (2005), varies with the number of indicators. A Consistency Ratio (CR) below 10% confirms statistically valid coefficients in vector $\overrightarrow{v}$, for index construction (Saaty 1987; Saaty 1990, 2006).

### Selecting the aggregation function

In this study, we evaluated two standard aggregation functions for sub-indices in multicriteria risk assessment (Nguyen et al. 2025): the weighted arithmetic and the weighted geometric means, as shown in Eqs. (10) and (11). We calculated the sensitivity of both aggregation methods and selected the more responsive one.

To compare the responsiveness of the weighted arithmetic and weighted geometric means used to aggregate the sub-indices, a structured sensitivity analysis was conducted, focusing on the two most influential factors identified by the F-AHP: the highest-weighted parameter (UVI) and the lowest-weighted parameter (OG). In this analysis, K_UVI_ and K_OG_ were perturbed by one-unit increments across their admissible ranges, while all other factors and their weights were held constant. The weighted geometric mean exhibited stronger risk discrimination, amplifying the impact of extreme UVI variations and more clearly penalizing combinations in which any single sub-index assumed a high-risk value. In contrast, the weighted arithmetic mean tended to smooth these extremes and produced less pronounced changes in the overall SEES. This behavior is desirable for a preventive risk indicator, since it ensures that critical excursions in a single environmental factor are not masked by more moderate conditions in the remaining dimensions, thereby yielding a more responsive and precautionary composite score.

From a theoretical standpoint, both arithmetic and geometric aggregation are widely used in multicriteria environmental and health-risk indices, including air quality and composite health-exposure scores. The arithmetic mean preserves linear compensation between criteria, allowing high values in one dimension to offset low values in another. In contrast, the geometric mean is more conservative by construction, reducing full compensation and increasing the influence of the worst-performing sub-index. In applications where extreme exposures in any single factor (e.g., UVR or fine particulate matter) are expected to play a critical role in health outcomes, the literature often recommends geometric aggregation as a way to avoid masking high-risk conditions, which aligns with the objectives of the SEES and with the results of our sensitivity analysis.

10 $$\mathrm{W}\mathrm{e}\mathrm{i}\mathrm{g}\mathrm{h}\mathrm{t}\mathrm{e}\mathrm{d}\mathrm{a}\mathrm{r}\mathrm{i}\mathrm{t}\mathrm{h}\mathrm{m}\mathrm{e}\mathrm{t}\mathrm{i}\mathrm{c}\mathrm{m}\mathrm{e}\mathrm{a}\mathrm{n}:\mathrm{S}\mathrm{E}\mathrm{E}\mathrm{S}=\sum_{1}^{\mathrm{n}}{\mathrm{v}}_{\mathrm{i}}{\mathrm{K}}_{\mathrm{i}}$$

11 $$\mathrm{W}\mathrm{e}\mathrm{i}\mathrm{g}\mathrm{h}\mathrm{t}\mathrm{e}\mathrm{d}\mathrm{g}\mathrm{e}\mathrm{o}\mathrm{m}\mathrm{e}\mathrm{t}\mathrm{r}\mathrm{i}\mathrm{c}\mathrm{m}\mathrm{e}\mathrm{a}\mathrm{n}:\mathrm{S}\mathrm{E}\mathrm{E}\mathrm{S}=\prod_{1}^{\mathrm{n}}{{\mathrm{K}}_{\mathrm{i}}}^{{\mathrm{v}}_{\mathrm{i}}}$$

Here, ${v}_{i}$ is the weight of the i factor; ${K}_{i}$ is the sub-index of the i factor, and the SEES is the proposed Skin Environmental Exposure Score (SEES).

To facilitate interpretation by readers from different disciplines, the main terms used in the SEES construction are summarized in Table 2:

**Table 2:** Key terminology

Term	Definition
Factor	A broad environmental determinant affecting skin health. Conceptual criterion or category grouping one or more related parameters (e.g., ultraviolet radiation, heat stress, air pollutants) used in the hierarchical F-AHP structure.
Parameter	Measurable environmental variable entering the model before normalization (e.g., UVI, pollutant concentration, thermal index) from which a sub-index is computed.
Sub-index (K_i_)	Dimensionless score representing the risk level associated with a single environmental factor (typically ranging 0–1 or 0–10) derived from the measured or estimated value of a specific parameter (e.g., UVR, O3, PM, OG), obtained after normalization of the corresponding raw variable. It quantifies the relative risk level associated with that single factor.
Weight (ν_i_)	A coefficient derived via the Fuzzy AHP process, representing the relative importance of a factor (and its associated sub-index) in contributing to the overall composite index.
SEES	The Skin Environmental Exposure Score: The final composite index, calculated by aggregating all weighted sub-indices (Ki) using the selected function.

### Risk index development

#### Risk factors related to the external skin exposome

The ${K}_{i}$ risk coefficients defined in this study are based on established environmental indices, which are detailed in this section. These indices serve as the foundational, quantitative input for our model, as they are the primary tools for publicly reporting conditions related to solar exposure, thermal discomfort, and air quality. For a comprehensive review, the governing equations and specific details for each index are available in the Supplementary Material.

UVR health risk is quantified using the Ultraviolet Index (UVI). The UVI, endorsed by the World Health Organization (WHO), represents more than a theoretical construct; it serves as a standardized, unitless measure with practical applications for evaluating erythema-inducing UVR risk at specific locations and times (WHO, 2002). The UVI scale directly correlates with erythemal irradiance, where 1 UVI unit equals 25 mW m^− 2^ of erythemal irradiance. Erythemal irradiance is calculated as the spectrally-weighted solar UV irradiance (between 280 and 400 nm) using the CIE erythema action spectrum for human skin (Mckinlay and Diffey, 1987). Higher UVI indicates greater potential for skin, hair, and ocular damage, with shorter exposure times required to cause harmful effects. The humidex index (H) was selected to assess heat-stress risk (Masterton and Richardson 1979). This index quantifies the perceived thermal sensation experienced by combining air temperature (T) and relative humidity (RH). Environment and Climate Change Canada (ECCC) states that a Humidex value below 20 indicates comfortable thermal environment, whereas values above 45 pose severe health risks.

Air Quality was evaluated using the Air Quality Index (AQI), which aggregates ground-level pollutant concentrations, including O_3_, PM, CO_2_, SO_2_, and NO_2_. The AQI is a standardized numerical scale used to communicate to the public how polluted the air currently is or how polluted it is forecast to become. The score for each pollutant is nonlinear, and AQI_i_ can be calculated separately for each component (i). It translates complex air quality data into a single, easy-to-understand number and color that indicates the level of health risk associated with breathing the air. Higher AQI values correspond to poorer air quality.

The assigned K_i_ risk coefficients, based on the categorized ranges of the environmental indices (UVI, H, and AQI_i_), are presented in Table 3. Consult Supplementary Material for further details on the full index scales and exposure risk categories.

**Table 3:** UVI, H, and AQIi thresholds and related Ki index

UVI	Humidex range (H)	AQI_i_ limits	K_i_
0–2	0–19	0–50	1
3–5	20–29	51–100	2
6–7	30–39	101–150	3
8–10	40–45	151–200	4
> 11	> 45	201–250	5
		251–300	6

#### Pairwise Hierarchical Levels (φ), weight assignment, and AHP application

The matrix Φ (Eq. 1) for the proposed index is constructed by performing pairwise comparisons between five key variables: UVI, H, O_3_, PM_2.5_, and a combined category for other gases (OG), where OG represents the aggregated AQI for the minor gases SO_2_ and NO_2_. Each element φ_ij_ of matrix Φ_SEES_ corresponds to the comparative value between variable pairs, with rows (i) and columns (j) following the order of the variables as listed: (1) UVI, (2) H, (3) O_3_, (4) PM_2.5_, and (5) OG.

12 $$\begin{array}{c}{{\Phi}}_{EEI}=\\\left(\begin{array}{ccc}(1,1,1)&(5,6,7)&\begin{array}{ccc}(\mathrm{5,6},7)&(5,6,7)&(9,9,9)\end{array}\\(\raisebox{1ex}{$1$}\!\left/\!\raisebox{-1ex}{$7$}\right.,\raisebox{1ex}{$1$}\!\left/\!\raisebox{-1ex}{$6$}\right.,\raisebox{1ex}{$1$}\!\left/\!\raisebox{-1ex}{$5$}\right.)&(1,1,1)&\begin{array}{ccc}(1,1,1)&(1,2,3)&(5,6,7)\end{array}\\\begin{array}{c}(\raisebox{1ex}{$1$}\!\left/\!\raisebox{-1ex}{$7$}\right.,\raisebox{1ex}{$1$}\!\left/\!\raisebox{-1ex}{$6$}\right.,\raisebox{1ex}{$1$}\!\left/\!\raisebox{-1ex}{$5$}\right.)\\(\raisebox{1ex}{$1$}\!\left/\!\raisebox{-1ex}{$7$}\right.,\raisebox{1ex}{$1$}\!\left/\!\raisebox{-1ex}{$6$}\right.,\raisebox{1ex}{$1$}\!\left/\!\raisebox{-1ex}{$5$}\right.)\\(\raisebox{1ex}{$1$}\!\left/\!\raisebox{-1ex}{$9$}\right.,\raisebox{1ex}{$1$}\!\left/\!\raisebox{-1ex}{$9$}\right.,\raisebox{1ex}{$1$}\!\left/\!\raisebox{-1ex}{$9$}\right.)\end{array}&\begin{array}{c}(1,1,1)\\(\raisebox{1ex}{$1$}\!\left/\!\raisebox{-1ex}{$3$}\right.,\raisebox{1ex}{$1$}\!\left/\!\raisebox{-1ex}{$2$}\right.,\raisebox{1ex}{$1$}\!\left/\!\raisebox{-1ex}{$1$}\right.)\\(\raisebox{1ex}{$1$}\!\left/\!\raisebox{-1ex}{$4$}\right.,\raisebox{1ex}{$1$}\!\left/\!\raisebox{-1ex}{$3$}\right.,\raisebox{1ex}{$1$}\!\left/\!\raisebox{-1ex}{$2$}\right.)\end{array}&\begin{array}{ccc}(1,1,1)&(1,2,3)&(5,6,7)\\(\raisebox{1ex}{$1$}\!\left/\!\raisebox{-1ex}{$3$}\right.,\raisebox{1ex}{$1$}\!\left/\!\raisebox{-1ex}{$2$}\right.,\raisebox{1ex}{$1$}\!\left/\!\raisebox{-1ex}{$1$}\right.)&(1,1,1)&(2,3,4)\\(\raisebox{1ex}{$1$}\!\left/\!\raisebox{-1ex}{$7$}\right.,\raisebox{1ex}{$1$}\!\left/\!\raisebox{-1ex}{$6$}\right.,\raisebox{1ex}{$1$}\!\left/\!\raisebox{-1ex}{$5$}\right.)&(\raisebox{1ex}{$1$}\!\left/\!\raisebox{-1ex}{$4$}\right.,\raisebox{1ex}{$1$}\!\left/\!\raisebox{-1ex}{$3$}\right.,\raisebox{1ex}{$1$}\!\left/\!\raisebox{-1ex}{$2$}\right.)&(1,1,1)\end{array}\end{array}\right)\end{array}$$

The φ weights assigned to each pairwise criterion reflect their relative importance but inherently carry uncertainty and subjectivity (Saaty 2005). To mitigate this, we adopted a harm-based scale prioritizing skin health risks. UVR holds the highest weight due to its well-documented carcinogenicity (IARC, 1992; Zhang et al. 2019), followed by thermal stress and surface ozone (Corrêa et al. 2021; Passeron et al. 2021). This hierarchy aligns with evidence linking UVR to the development of skin cancer (Chang and Chen 2011; Teng et al. 2021; Schalka and Corrêa 2024), underscoring its primacy in our assessment.

Thermal stress, though secondary, significantly potentiates skin damage by activating heat shock proteins, altering signaling pathways, and increasing cutaneous permeability and blood flow, factors that exacerbate UVR-induced carcinogenesis (Van Der Leun et al. 2008). Among air pollutants, ozone (O₃) poses the most significant risk, inducing lipid peroxidation in the epidermis and triggering inflammatory responses (Thiele et al. 1997), while particulate matter (PM₂.₅) contributes to oxidative stress (Baudouin et al. 2002; Drakaki et al. 2014). Although PM₂.₅’s effects are less severe than UVR or O₃, its inclusion is critical given its role as a proxy for local nanoparticle pollution (ultrafine particles were excluded due to data limitations).

Finally, other gases (OG; NO₂, and SO₂) were assigned the lowest weights due to their comparatively minor skin impacts and higher uncertainty. Future iterations could incorporate volatile organic compounds (VOCs), polycyclic aromatic hydrocarbons (PAHs), and ultrafine particles if data become available. This tiered weighting system balances known risks with practical constraints, offering a scalable framework for environmental skin health assessments.

#### The skin environmental exposure score (SEES)

The SEES integrates the cumulative effects of environmental exposures using an aggregation function that maintains sensitivity across each factor’s full dynamic range (Khouri and Al-Moufti 2022). Sensitivity analyses reveal that the weighted geometric mean (20.7% sensitivity) outperforms the arithmetic mean (10.2%), as it both preserves the predefined hierarchical weighting of factors and more effectively captures their multiplicative and synergistic interactions. The detailed results of these sensitivity analyses are available in the supplementary material. Unlike the arithmetic mean, which dilutes variations uniformly and disproportionately attenuates the influence of high-priority factors like UV radiation (the dominant driver of extrinsic skin damage), the geometric mean’s nonlinear properties ensure that critical risks retain their proportional impact in the aggregated index. This approach aligns with the biological reality of compounded environmental stressors while respecting the SEES’s foundational risk hierarchy.

The SEES is formulated as a weighted geometric combination of exposure risk coefficients (K_i_), where the hierarchical weighting is determined by the eigenvector $\overrightarrow{v}$, provided by Eq. (6): $$\overrightarrow{v}=\left(\begin{array}{c}0.58\\0.15\\\begin{array}{c}0.15\\0.08\\0.04\end{array}\end{array}\right)$$. This yields:

13 $$\begin{array}{c}\mathrm{SEES}=C\prod v_i^{K_i}=\\C\left({0.58}^{K_{UV}}\ast{0.15}^{K_H}\ast{0.15}^{K_{O3}}\ast{0.08}^{K_{PM}}\ast{0.04}^{K_{OG}}\right)\end{array}$$

Where C is a normalization factor scaling the index to a 0-1000 range (Han et al. 2011):

14 $$\mathrm{C}=\frac{\mathrm{S}\mathrm{E}\mathrm{E}\mathrm{S}-{\mathrm{S}\mathrm{E}\mathrm{E}\mathrm{S}}_{\mathrm{m}\mathrm{i}\mathrm{n}}}{{\mathrm{S}\mathrm{E}\mathrm{E}\mathrm{S}}_{\mathrm{m}\mathrm{a}\mathrm{x}}-{\mathrm{S}\mathrm{E}\mathrm{E}\mathrm{S}}_{\mathrm{m}\mathrm{i}\mathrm{n}}}*1000$$

SEES_min_ = 1.00 and SEES_max_ = 5.25 represent the theoretical minimum and maximum risk conditions, corresponding to all K_i_ values at their lower and upper bounds, respectively.

The weighting scheme’s statistical consistency was verified through the consistency ratio (CR). Beyond its numerical value, the CR has direct practical implications for interpreting the reliability of the weight set. In this study, the maximum eigenvalue λ_max_ = 5.22 yields a consistency index (CI) of 5.5% and a CR of 4.9%, which is well below the conventional CR threshold of < 10%. This indicates that the harm-based pairwise judgments used to prioritize UVR, H, O3, PM2.5, and OG are internally coherent: if one factor is judged more influential than another, these preferences are not systematically contradicted elsewhere in the comparison matrix. For readers less familiar with F-AHP, a low CR indicates that the resulting eigenvector $\overrightarrow{v}$ (Eq. 6) can be interpreted as a stable and logically consistent representation of relative risk contributions. In contrast, a CR exceeding the 10% threshold would signal that the expert judgments should be revisited, clarified, or re-elicited before using the weights in index construction.

Despite the mathematical robustness of the SEES formulation and the satisfactory performance of the F-AHP framework, the resulting weights and risk coefficients are inherently context dependent. In contrast to standardized indicators such as the UVI and the AQI, which are not regionally recalibrated but instead provide a normalized description of a particular environmental condition at a given place and time, the SEES is designed as a composite risk score that integrates multiple exposures into a single scale. As a consequence, the current hierarchy, in which UVR dominates, followed by thermal stress, O_3_, PM, and OG, captures a broadly applicable, harm-based prioritization that is expected to remain valid across many mid-latitude urban environments and under a wide range of typical conditions. Regional recalibration of weights is therefore not strictly necessary for most practical applications, provided that the local exposure profiles fall within the domain of variability for which the current hierarchy was conceived. Nevertheless, in settings with markedly different UV regimes, pollutant mixtures, or thermal profiles (e.g., high-altitude regions, heavily industrialized areas, or climates with extreme heat and humidity), or when new epidemiological evidence indicates substantially different relative impacts of each factor, a targeted recalibration procedure, ideally involving local experts and updated exposure or health data, may be warranted to ensure that the SEES maintains its relevance and accuracy. Periodic review of the pairwise judgments and resulting priorities, particularly under rapid climate or urban changes, is recommended as good practice rather than as a strict requirement for routine SEES use.

## Results and discussion

In this section, results and discussion are presented jointly so that the spatial and temporal patterns of the SEES can be interpreted immediately in light of their underlying environmental drivers and public health implications.

### Sensitivity test and the SEES scale

The SEES classifies environmental exposure risks into seven distinct levels of health impact: none, low, moderate, elevated, high, severe, and extreme. This classification system was developed through a rigorous three-stage process.

First, we performed comprehensive sensitivity analyses across all possible combinations of exposure scenarios using the risk coefficients K_i_. The UVI scale was adopted as the reference framework for defining risk categories, reflecting both its predominant weighting (58%) in the SEES and its well-established correlation with dermatological damage. Second, quantitative thresholds between risk levels were systematically determined by evaluating individual variability in each environmental factor (UVR, heat stress, ozone, PM2.5, and other gases), their synergistic effects on cutaneous health, and their cumulative damage potential across combined exposure scenarios. Finally, the proposed thresholds were validated against known biological thresholds for UV-induced skin damage, epidemiological data on multi-stressor dermatological effects, and clinical benchmarks for environmentally driven skin pathologies.

The resulting classification system (presented in Table 4) provides a robust, biologically grounded framework for assessing environmental health risks. This approach offers significant improvements over conventional single-stressor assessment methods by anchoring the SEES categories to established UV risk paradigms while accounting for combined exposure effects.

**Table 4:** Thresholds and risk classification based on SEES values

SEES thresholds	Risk factor	Color (RGB code)
0–65	None	Green (146, 208, 80)
66–139	Low	Light Yellow (251, 236, 93)
140–229	Moderate	Yellow (255, 255, 0)
230–369	Worrying	Orange (255, 140, 0)
370–489	High	Light Red (255, 70, 0)
490–534	Very High	Red (255, 0, 0)
530–794	Severe	Purple (128, 0, 128)
795–1000	Extreme	Brown (85, 39, 7)

**Fig. 2:**
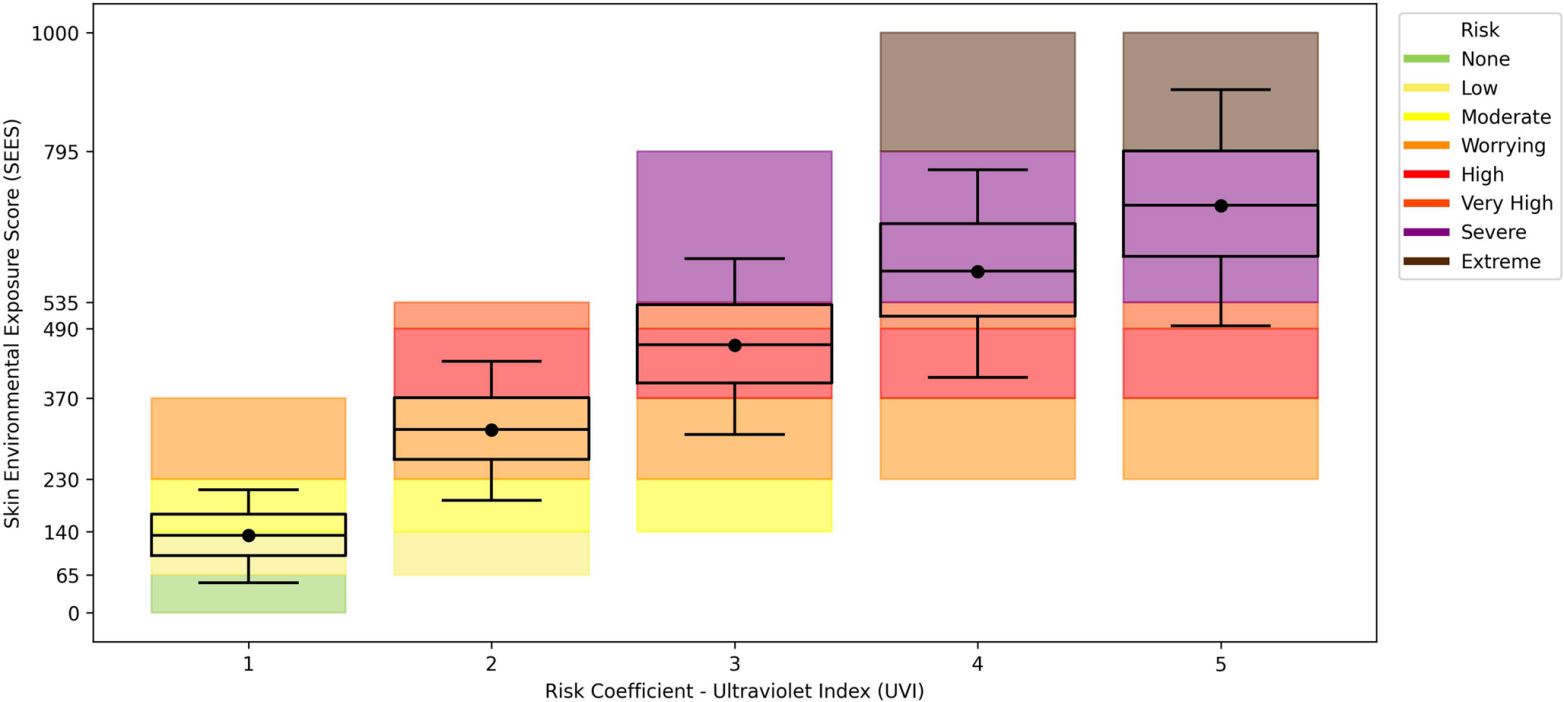
Comparative analysis between UVI risk variation (K_UV_) and SEES risk factors

Figure 2 shows the distribution of SEES values from sensitivity analysis across UVR risk coefficients (K_UV_ = 1–5), with color-coded bands corresponding to the risk categories (see Supplementary Material). The visualization demonstrates how equivalent K_UV_ values can yield divergent SEES classifications due to modulating effects from co-occurring environmental stressors, highlighting the index’s capacity to capture multifactorial exposure dynamics.

The “no risk” category (K_UV_ = 1, equivalent to UVI < 2) is the only scenario in which photoprotection is unnecessary according to WHO (2002) guidelines. While this category may coincide with mild thermal discomfort or moderate pollution, these conditions alone pose negligible risks to skin health for most populations. However, our analysis reveals that even at K_UV_ = 1, elevated thermal discomfort or pollution can escalate the risk to “Low”, demonstrating the SEES’s sensitivity to combined environmental effects.

“Moderate” risk emerges when intermediate UVR exposure (K_UV_ ≤ 2) coincides with substantial thermal discomfort or pollution (K_i_ ≥ 2, where i = H or O_3_). While individually non-critical, such combinations amplify UV-induced damage synergistically (Van Der Leun et al. 2008; De Vecchi et al. 2019), highlighting the SEES’s ability to detect multifactorial risks that traditional single-stressor metrics would miss.

The upper-risk tiers (“Worrying” to “Extreme”) reflect dangerous intersections of high UVR (3 ≤ K_UV_ ≤ 5) with escalating thermal and pollution stressors. Notably, K_UV_ = 5 scenarios consistently reach “Very High” or “Extreme”, preserving UVR’s dominant role in skin damage. Climate projections (Yamamoto et al. 2024) suggest these high-risk combinations will become increasingly prevalent, positioning the SEES as a critical tool for climate health preparedness.

This advancement addresses the key limitations of conventional UVI scales, which were primarily calibrated for temperate zone contexts (Zaratti et al. 2014). Integrating multiple environmental factors while maintaining skin-type neutrality, the SEES provides a more globally relevant framework, particularly for tropical regions with frequent and intense combined exposures.

### Climatic database for applying the SEES

#### Global database

We evaluated the SEES globally for solstice and equinox conditions using two leading reanalysis datasets: NASA’s Modern-Era Retrospective Analysis for Research and Applications, Version 2 (MERRA-2), and ECMWF Reanalysis version 5 (ERA5). The MERRA-2 dataset (Gelaro et al. 2017), with a spatial resolution of 0.5° × 0.625°, provided key atmospheric variables including surface mass concentrations SO_2_ (SO2SMASS; kg m − 3), Black Carbon (BCSMASS; kg m − 3), Organic Carbon (OCSMASS; kg m-3), SO_4_ (SO4SMASS; kg m-3), Dust - PM 2.5 (DUSMASS25; kg m-3), and Sea Salt - PM 2.5 (SSSMASS25; kg m-3). Additionally, we utilized MERRA-2’s aerosol optical depth (AODANA, dimensionless), ozone mass mixing ratio (O3; kg kg-1), and total column ozone (TO3; Dobson Units). These data were processed through the Tropospheric Ultraviolet and Visible (TUV) Radiation Model (Madronich, 1997) to derive ultraviolet index (UVI) values for SEES calculation.

The higher-resolution ERA5 reanalysis (Hersbach et al. 2020), featuring a 0.25° × 0.25° global grid, supplied complementary meteorological variables, including 2-meter air temperature (t; K) and relative humidity (r; %). By combining these datasets, we established the risk coefficients (K_i_) necessary for SEES computation, leveraging MERRA-2’s comprehensive pollutant data and ERA5’s refined atmospheric parameters. This integrated approach enabled a robust assessment of combined environmental exposures across different spatial scales and temporal conditions.

#### Local database

This study utilized air quality and meteorological data from monitoring stations in São Paulo and Rio de Janeiro during thermal stress or air pollution events. In São Paulo, measurements were acquired from the CETESB-operated Parque Dom Pedro II station (23.5°S, 46.6°W). At the same time, Rio de Janeiro were collected at the Adalgisa Nery station (22.9°S, 43.0°W) under DataRio’s Air Quality Monitoring Program. Both stations provided hourly records of O_3_, PM_2.5_, NO_2_, and SO_2_ concentrations, along with air temperature and relative humidity measurements.

Ultraviolet index (UVI) data were obtained from the Center for Weather Forecasting and Climate Studies (CPTEC/INPE) website, derived from NASA’s Aura satellite Ozone Monitoring Instrument (OMI). The OMI sensor quantifies ground-level UVR by precisely measuring backscattered sunlight, providing daily global coverage at high spatial resolution. This satellite-based approach complements the in-situ air quality measurements, enabling a comprehensive assessment of environmental exposure factors.

The SEES applications presented here rely on a combination of satellite products, reanalysis data (MERRA‑2, ERA5), and fixed monitoring stations in São Paulo and Rio de Janeiro, which introduces limitations related to sensor spatial coverage, representativeness, and temporal sampling. Sparse ground-based networks may fail to capture intraurban gradients in pollution and thermal stress. In contrast, hourly or daily sampling can miss short-lived peak conditions relevant to acute skin exposure.​.

To mitigate these constraints, future implementations of SEES can: (i) use high‑resolution climatologies derived from long-term reanalysis and satellite records to estimate background patterns of UVR, heat, and pollutants; (ii) incorporate numerical modeling (e.g., chemical transport and urban climate models) to resolve fine-scale spatial variability and fill gaps between stations; and (iii) assimilate emerging low‑cost sensor networks, where available, to refine local exposure estimates. Such strategies would enhance SEES’ robustness in regions with limited monitoring infrastructure and support more spatially detailed risk assessments.

It is important to note that the SEES, as applied here, represents area‑averaged environmental conditions and does not yet resolve intraurban or population-level variability in vulnerability. Differences in socioeconomic status, occupational profiles, housing quality, and access to sun protection (e.g., shade, clothing, sunscreen, and air‑conditioned environments) can substantially modulate the actual risk experienced by individuals and communities exposed to the same SEES category. Consequently, while the SEES is a valuable tool for identifying spatiotemporal hotspots of environmental exposure, its use in public policy should be complemented by social and behavioral data to prioritize interventions in the most vulnerable populations.

### Applications

#### Seasonal and hourly global variations of the skin environmental exposure score (SEES) and interpretation of combined exposure risks

**Fig. 3:**
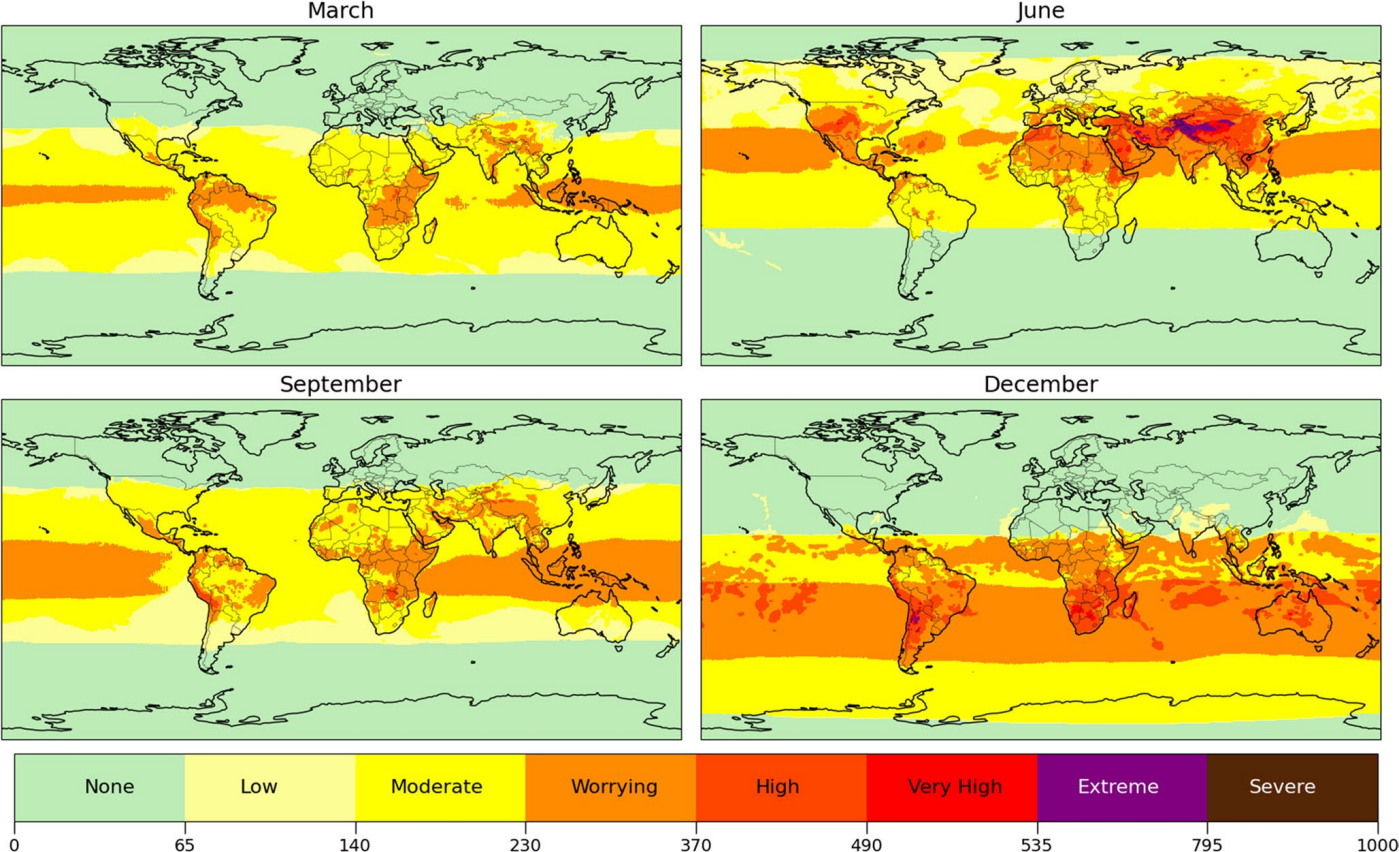
Seasonal variation of skin environmental exposure score (SEES) at 9 am on Equinoxes and Solstices in 2022

As established, the SEES exhibits pronounced diurnal and seasonal variability driven primarily by UVR, though concurrent environmental factors significantly modify exposure risks. We evaluated these patterns using MERRA-2 and ERA5 data for 2022, through representative temporal snapshots. The global patterns presented below are derived from the harm‑based weighting scheme introduced in Sect.  2.3.3, which discusses the regional applicability and the possible recalibration of weights and thresholds. Our analysis reveals distinct spatial patterns during solstices and equinoxes, as shown in Figs. 3 (9 am local time) and 4 (local noon). Tropical and subtropical regions consistently exhibited the highest health risks, with morning SEES values frequently reaching low-to-elevated levels during summer and equinox periods. Notably, southern tropical latitudes maintained elevated-to-high risk classifications across most latitudes, even in the early morning hours, primarily due to persistent heat stress.

Three principal spatial patterns emerged from this global analysis. First, pollution-dominated extreme SEES values during June mornings correlated strongly with anthropogenic emissions in industrialized regions like China, India, and parts of the United States, where concurrent heat waves further exacerbated risks. Second, temperature-driven SEES elevations were particularly evident in December across South America, southern Africa, and central Australia, coinciding with seasonal temperature maxima. These patterns were complemented by dust-related increases in PM_2.5_ over northern Africa and southern Asia, associated with Saharan transport events (Vasilatou et al. 2017). Third, high-altitude regions such as the Andes showed persistently elevated SEES values resulting from the combined effects of enhanced UVR, reduced water vapor, altitude-dependent ozone increases, and seasonal temperature extremes (Rivas et al., 2023; Salas et al. 2017).

These findings underscore how non-UVR environmental parameters can critically modify baseline exposure risks, particularly during periods of meteorological extremity. The SEES’s integrated methodology successfully captures these multifactorial interactions that traditional UV-focused indices would overlook, providing a more comprehensive assessment of environmental health risks across diverse geographic and climatic contexts.

**Fig. 4:**
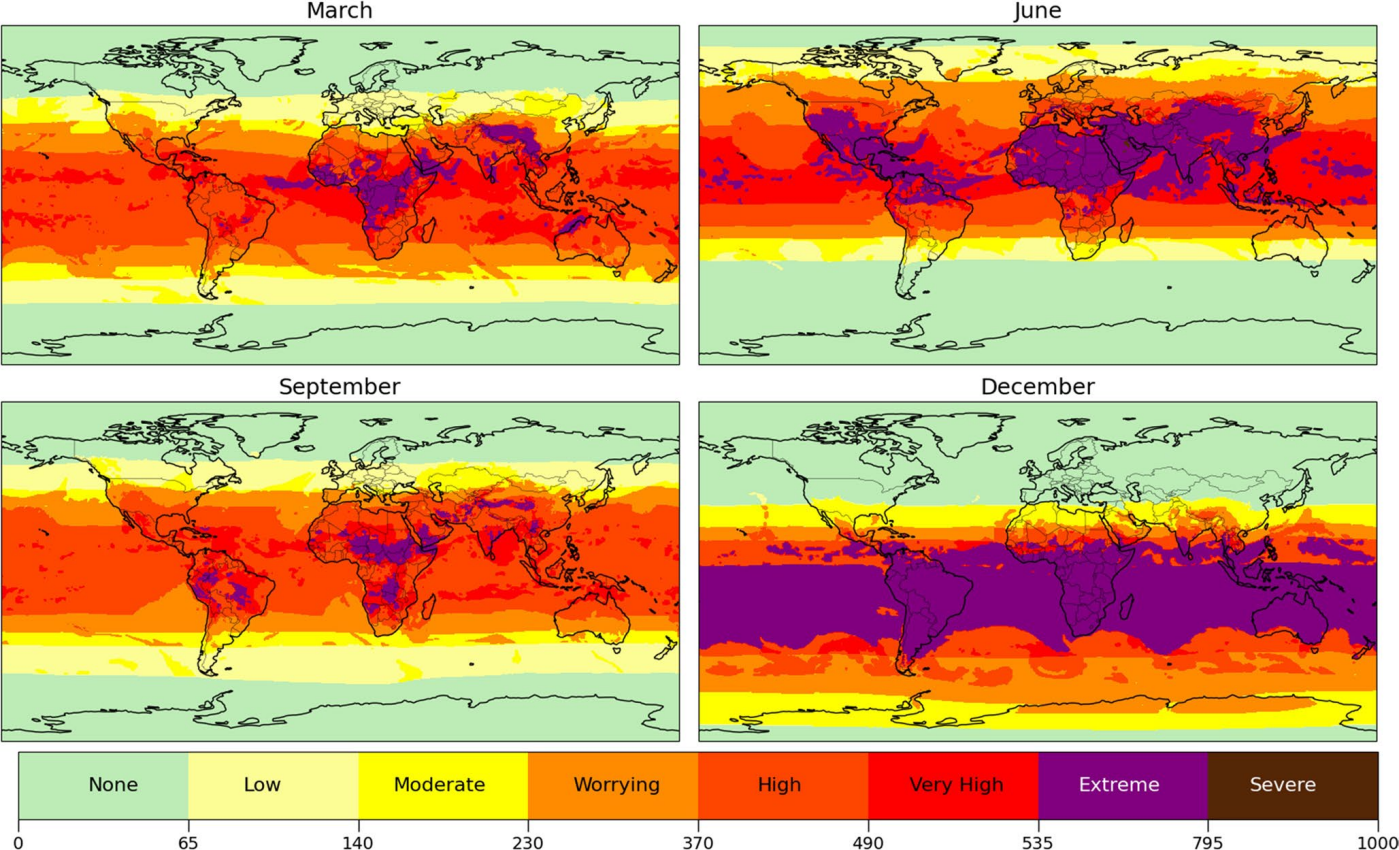
Seasonal variation of skin environmental exposure score (SEES) at noon on Equinoxes and Solstices in 2022

Figure 4 shows the SEES values at local solar noon during solstices and equinoxes, capturing peak solar radiation conditions when UVR reaches its daily maximum. As expected, tropical and subtropical regions consistently demonstrate high-to-extreme SEES values, with severe classifications emerging where additional environmental stressors compound UVR exposure. From a public health perspective, these patterns indicate that even moderate SEES levels may correspond to conditions requiring strengthened sun-protection and heat-mitigation guidelines in densely populated urban areas.

The equinoxes reveal particularly noteworthy patterns in central Africa and Southeast Asia, where combined heat stress and air pollution drive SEES to its highest levels. In African regions, elevated temperatures coupled with high humidity during rainy seasons (Yang et al. 2015; Philippon et al. 2016) interact with persistent PM₂.₅ pollution from urban emissions, dust transport, and open burning (Petkova et al. 2013; Fayiga et al. 2017; Jiying et al. 2023). Climate projections suggest these risks will intensify as heatwave frequency and duration increase and precipitation patterns shift across Africa (Diedhiou et al., 2018; Fotso-Nguemo et al. 2022).

Summer months show distinct regional SEES amplifications. The Middle East experiences SEES elevation driven by extreme temperatures, and Southeast Asian SEES is influenced by monsoon-driven aerosol dispersion, which modifies surface temperatures and circulation patterns (Lau et al. 2006; Bollasina et al. 2014). Western Asia’s exceptionally high PM_2.5_ concentrations (K_PM_ > 4) further exacerbate June’s SEES in South Asia (Singh et al. 2019).

Temperate regions exhibit substantial hemispheric seasonal variability in SEES, where air pollution and thermal discomfort significantly modify baseline UVR risks. These patterns highlight the critical need for region-specific preparedness strategies that account for the compound nature of environmental exposures captured by the SEES framework. Besides, these patterns also suggest that reliance on UV-only metrics may underestimate risk in regions where heat stress and air pollution substantially amplify cutaneous damage. Taken together, these results indicate that SEES can distinguish between UV‑driven and pollution‑driven events, providing more specific guidance for targeted interventions.

#### Skin Environmental Exposure Score (SEES) assessment and interpretation during pollution and heat stress episodes

**Fig. 5:**
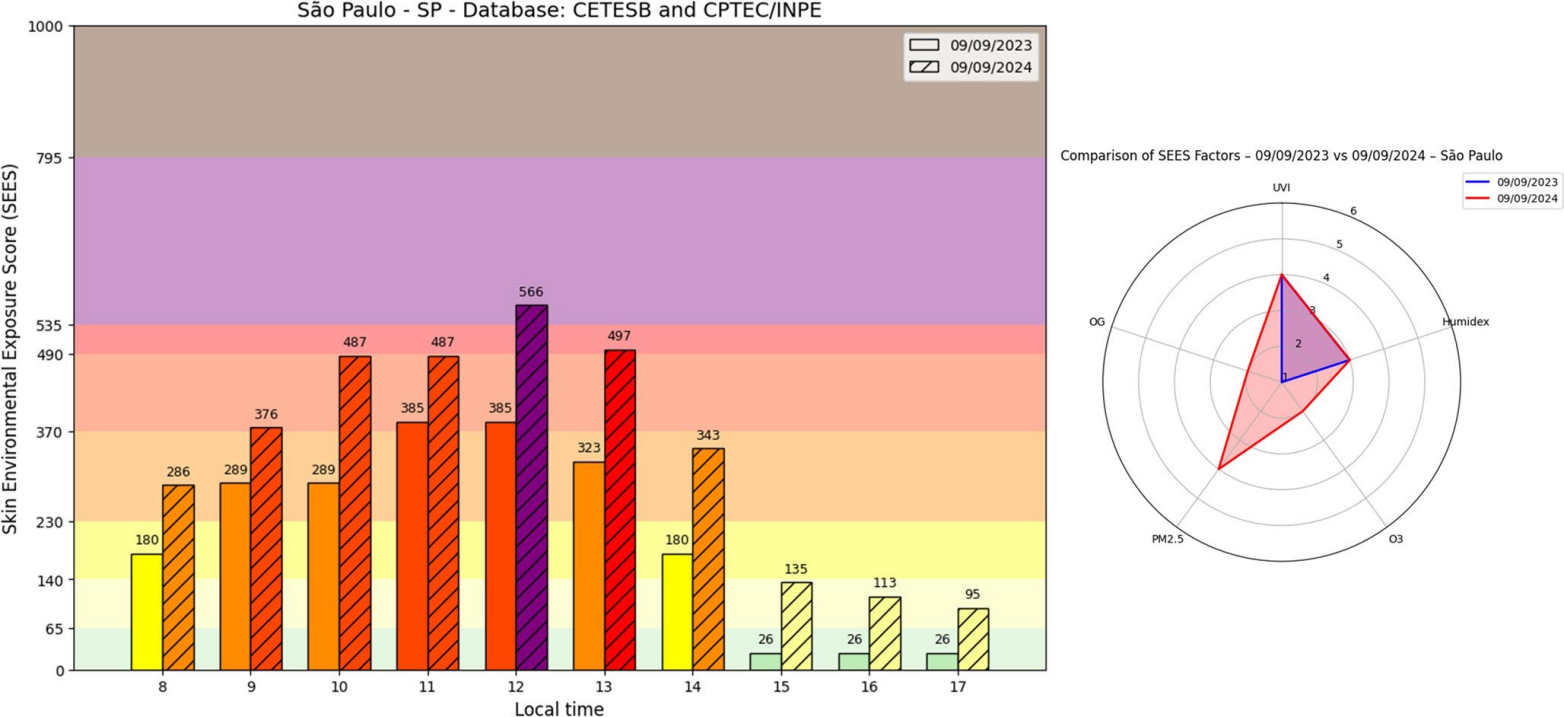
Hourly variation of the skin environmental exposure score (SEES) on September 9 for the years 2023 and 2024 in São Paulo, SP. The bar chart (left) displays SEES values from 8 am to 5 pm, with background colors indicating risk categories. The radar chart (right) compares the contribution of individual environmental factors (UVI, Humidex, O_3_, PM_2.5_, and OG) to the SEES maximum observed at noon each year

Figure 5 illustrates the diurnal variation of the SEES in São Paulo, Brazil, on September 9 during 2023 and 2024. The index reached significantly higher levels in 2024 (SEES = 633, extreme category) compared to 2023 (SEES = 483, high range), demonstrating the substantial impact of extreme environmental conditions on exposure risks.

According to IQAir, the exceptional SEES peak in 2024 coincided with record-breaking air pollution levels that temporarily ranked São Paulo as the world’s most polluted city, receiving widespread media coverage (CNN 2024; G1 2024). CETESB reported that August-September 2024 saw unprecedented pollutant concentrations in the São Paulo Metropolitan Region (RMSP), driven by multiple factors, including extensive wildfires and persistent atmospheric stability that trapped PM_2.5_ and O₃ near the surface (CETESB, 2024).

The radar chart in Fig. 5B reveals distinct compositional differences in SEES between the two years. While 2023 (highlighted in blue) showed relatively balanced contributions from all factors with expected UVI and H dominance during peak sunlight hours, 2024 (in red) exhibited dramatically elevated PM_2.5_ and UVI impacts, followed by H, O_3_, and OG. This shift reflects both the acute air quality crisis and the growing influence of pollution on cutaneous health risks.

Notably, while SEES typically peaks at noon due to maximum solar irradiance, these results demonstrate how extreme meteorological events (heat waves, thermal inversions) and pollutant accumulation can substantially modify both the magnitude and temporal profile of exposure risks. The 2024 episode serves as both a specific public health alert and a concerning indicator of deteriorating urban environmental conditions, with significant implications for skin health in megacities facing increasing climate extremes. This finding reinforces the interpretation that high SEES values in tropical and subtropical regions are not solely a consequence of UVR intensity but emerge from the compounded effects of heat and air pollutants.

**Fig. 6:**
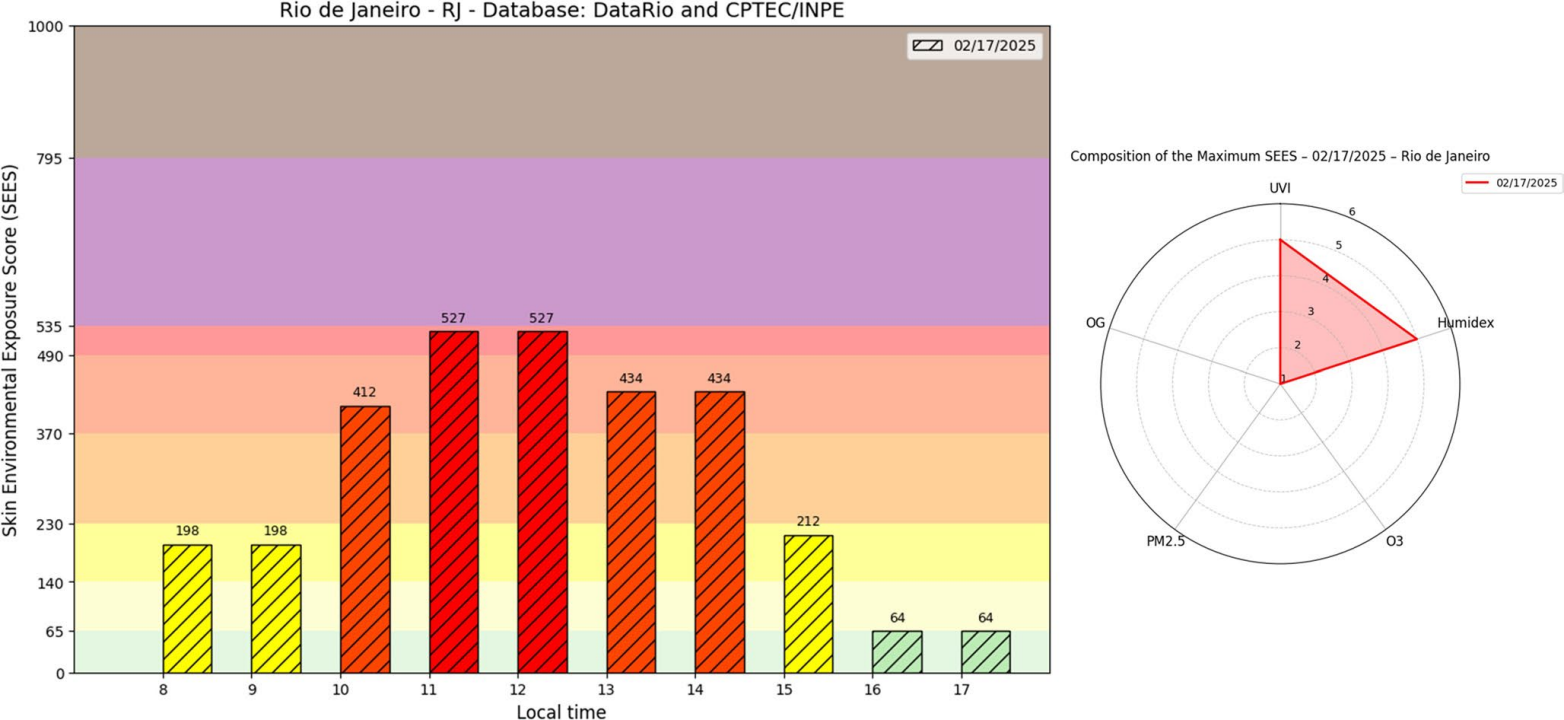
Hourly variation of the skin environmental exposure score (SEES) on February 17, 2025, in Rio de Janeiro, RJ. The bar chart (left) displays SEES values from 8 am to 5 pm, with background colors indicating risk categories. The radar chart (right) compares the contribution of individual environmental factors (UVI, Humidex, O_3_, PM_2.5_, and OG) to the SEES maximum observed at noon

Figure 6 demonstrates the temporal evolution of the SEES in Rio de Janeiro during a record-breaking heat event documented by national media (COR 2025; G1 2025). While peak SEES values reached the “Very High” category near solar noon, SEES remained elevated at “Moderate” to “Very High” levels from 8 am to 3 pm local time, indicating prolonged critical exposure conditions throughout daylight hours.

Analysis of the SEES composition via a radar chart reveals this event was driven exclusively by extreme heat stress (very high H) and (UVI), without significant contributions from air pollutants. Despite the absence of exacerbated pollution levels, the synergistic interaction between elevated temperature and UVI created particularly adverse conditions for cutaneous health.

These findings highlight an emerging pattern of climate-related health risks, where traditional midday exposure warnings may underestimate cumulative daily damage. As global warming and urban heat island effects intensify, such extended exposure events are projected to increase in frequency, positioning the SEES as a crucial tool for both real-time public health advisories and long-term urban climate adaptation strategies. The Rio de Janeiro case study exemplifies how even without extreme pollution, combined thermal and radiative stresses can sustain dangerous exposure windows that demand revised protection guidelines.

In both São Paulo and Rio de Janeiro, SEES values reflect the harm‑based hierarchy defined in Sect.  2.3.3; that section also discusses how this weighting scheme can be adapted or recalibrated for other environmental or demographic contexts, which is critical when extending these applications beyond the present case studies.

The comparative assessment of extreme events in São Paulo and Rio de Janeiro demonstrates SEES’s robust capacity to capture distinct environmental risk profiles that affect cutaneous health. During heatwave conditions characterized by clear skies and atmospheric stability, typically associated with lower pollutant concentrations, the SEES effectively identified elevated risks driven primarily by UVR and heat stress. Conversely, the score accurately reflected risk amplification predominantly caused by high PM_2.5_ concentrations in extreme pollution episodes.

In practical terms, the contrasting profiles observed in São Paulo and Rio de Janeiro illustrate how SEES can inform differentiated public health responses to pollution episodes versus extreme heat events. This dual sensitivity positions the SEES as a uniquely versatile tool for environmental health monitoring, capable of generating scenario-specific alerts tailored to different vulnerable populations. By accounting for both climatic and pollution-related stressors simultaneously, this score provides a comprehensive framework that surpasses conventional single-factor risk assessments. These attributes position SEES as a strategic tool for developing targeted public health policies and preventive strategies amid of increasing environmental challenges.

## Final considerations

The Skin Environmental Exposure Score (SEES) introduced in this study provides an integrated quantification of environmental risks to skin health by combining UVR, air pollution, and thermal stress into a single metric based on F-AHP. The SEES addresses critical gaps in existing environmental indices, which traditionally assess these factors in isolation. The hierarchical weighting scheme prioritizes UVR due to its well-documented carcinogenicity, followed by ozone, heat stress, and particulate matter, ensuring that the score reflects their relative dermatological harm and captures synergistic interactions between stressors in real-world exposure conditions.

Globally, the SEES identifies high-risk conditions in tropical and subtropical areas where pollution and heat exacerbate UVR effects, as well as in urban centers with elevated PM_2.5_ and O₃ levels. Seasonal and diurnal analyses reveal that extreme SEES values tend to occur during summer and around solar noon, with particularly critical situations over regions such as the Middle East, South Asia, and the Andes, while Brazilian case studies in São Paulo and Rio de Janeiro show the score’s sensitivity to both pollution-dominated episodes and intense heatwaves, underscoring its versatility for localized risk assessment. From a risk management perspective, the persistence of “Moderate” to “High” SEES levels outside the traditional midday peak supports the need to revise protection messages that focus only on a narrow time window.

The SEES methodology, supported by consistency checks and sensitivity analyses, provides a robust basis for public health advisories and climate adaptation strategies, enabling scenario-specific alerts for vulnerable populations and informing responses to the combined effects of climate change and urbanization on skin health. Future research should investigate long-term SEES trends under different climate scenarios and extend the framework to incorporate additional pollutants, such as VOCs and ultrafine particles, as suitable data becomes widely available.

By bridging environmental and dermatological perspectives, the SEES establishes a scalable, evidence-based framework to guide targeted protective measures and mitigate multifactorial skin damage. Its integration into routine monitoring systems could support real-time public health interventions and environmental education campaigns, particularly in tropical and highly urbanized regions, through intuitive communication tools such as color-coded risk bands for agencies, schools, and media outlets. In the longer term, SEES-based climatologies may help identify chronic exposure hotspots, assess the co-benefits of mitigation policies, and design focused strategies for at-risk populations, complementing existing UV and air quality indices with a more holistic view of skin-relevant environmental exposures.

## Supplementary Information

Below is the link to the electronic supplementary material.

Supplementary Material 1

## Data Availability

The datasets analyzed during the study and described in Sect.  2.6 are free and in the public domain.
