# Supplementary material for: A global score for skin environmental exposure: integrating UV radiation, heat, and pollution via fuzzy analytic hierarchy process
Source: Int J Biometeorol. 2026 Apr 13;70(4):117. doi: 10.1007/s00484-026-03145-0 (PMC13076557; doi:10.1007/s00484-026-03145-0)
Supplement: Supplementary file 1 — Supplementary Material 1 [file 484_2026_3145_MOESM1_ESM.docx]

**Supplementary material**

The Ultraviolet Index (UVI) is derived from erythemal irradiance (S), which is the integral of spectral solar irradiance (I_λ_) weighted by the CIE erythema action spectrum (ε_λ_) over the wavelengths 280 to 400 nm. A conversion factor of 40 is applied to S to define the dimensionless UVI scale, where 1 UVI unit corresponds to an erythemal irradiance of 25 mW m⁻²:

$UVI=40\times S$ (A.1)

$where S=\int_{280 nm}^{400 nm} \varepsilon_{\lambda}I_{\lambda}d\lambda$ (A.2)

The internationally recognized UVI classification and its associated health risk categories are presented in Table A.1.

**Table A.1** – Ultraviolet index scale and exposure categories (WHO, 2002)

| **UVI** | **Exposure category** |
| --- | --- |
| 0 – 2 | Low |
| 3 – 5 | Moderate |
| 6 – 7 | High |
| 8 – 10 | Very High |
| > 11 | Extreme |

The Humidex (H) is an index designed to quantify the perceived temperature for the average person by combining the air temperature (T) with the discomfort caused by humidity. It is calculated using equation A.3:

$H=T+ \left( \frac{5}{9} \right)\left[ \left( e_{s}\frac{\mathrm{RH}}{100} \right)-10 \right]$ (A.3)

This formula translates the physiological effect of humidity into an equivalent temperature sensation. The core of the humidity component is the term $e_{s}= 6,105 x e^{(17,27.T)/(237,7+T)}$. This is a standard empirical formula (the Magnus-Tetens formula) to calculate the saturation vapor pressure (in millibars) at a given air temperature T (in degrees Celsius). This represents the maximum amount of water vapor the air can hold at that temperature. The actual vapor pressure is calculated by multiplying $e_{s}$ by the relative humidity $\left( \frac{RH}{100} \right)$. The term [actual vapor pressure – 10] generates a humidity-based discomfort value. This value is then scaled by the factor (5/9) to convert it from metric units into a temperature-like number (degrees Celsius). Finally, this "perceived temperature due to humidity" is added to the actual air temperature T. A higher actual vapor pressure (from high temperature and/or high relative humidity) leads to a higher Humidex value, indicating hotter and more oppressive conditions that feel warmer than the actual air temperature. Table A.2 summarizes the risk coefficients associated with each H category (ECCC,2016):

**Table A.2** – Humidex range scale (ECCC, 2016)

| **Humidex range (H)** | **Degree of Comfort** |
| --- | --- |
| 0 – 19 | None |
| 20 – 29 | Little discomfort |
| 30 – 39 | Some discomfort |
| 40 – 45 | Great discomfort |
| > 45 | Dangerous |

Following the United States Environmental Protection Agency (EPA, 2018), the AQI is computed as:

$\mathrm{AQI}_{i}= \frac{I_{\mathrm{Hi}}-I_{\mathrm{Lo}}}{C_{\mathrm{Hi}}-C_{\mathrm{Lo}}}* \left( C_{i}-C_{\mathrm{Lo}} \right)+I_{\mathrm{Lo}}$ (A.4)

Where *i* represents a specific pollutant; *C_i_* is the monitored concentration of the pollutant; *C_Hi_* and *C_Lo_* are the upper and lower classes breakpoints concentrations for *C_i_*, respectively; and *I_Hi_* and *I_Lo_* are the index breakpoints corresponding to *C_Hi_* and *C_Lo_*, respectively. Table A.3 shows AQI pollutant’s risk coefficients and associated parameters.

**Table A.3** – Air Quality Index (AQI) threshold limits (EPA, 2018)

| **AQI_i_ limits** | |  | **O_3_ (ppm)**  **8-hour** | | **PM_2.5_** **(μg/m³)**  **24-hour** | | **SO_2_ (ppb)**  **1-hour** | | **NO_2_ (ppb)**  **1-hour** | |
| --- | --- | --- | --- | --- | --- | --- | --- | --- | --- | --- |
| **I_Lo_** | **I_Hi_** | **Levels of health concern** | **C_Lo_** | **C_Hi_** | **C_Lo_** | **C_Hi_** | **C_Lo_** | **C_Hi_** | **C_Lo_** | **C_Hi_** |
| 0 | 50 | Good | 0 | 0.054 | 0 | 12 | 0 | 35 | 0 | 53 |
| 51 | 100 | Moderate | 0.055 | 0.070 | 12.1 | 35.4 | 36 | 75 | 54 | 100 |
| 101 | 150 | Unhealthy^*^ | 0.071 | 0.085 | 35.5 | 55.4 | 76 | 185 | 101 | 360 |
| 151 | 200 | Unhealthy | 0.086 | 0.105 | 55.5 | 150.4 | 186 | 304 | 361 | 649 |
| 201 | 300 | Very unhealthy | 0.106 | 0.200 | 150.5 | 250.4 | - | - | 650 | 1.249 |
| 301 | 500 | Hazardous | - | - | 250.5 | 500.4 | - | - | 1.250 | 2.049 |

* Unhealthy for sensitive group

*i* can be O_3_, PM_2.5_, SO_2_ or NO_2_.

**Table A.4** – Sensitivity evaluation results of the two aggregation functions

| Factors | K_i_ | Weighted  arithmetic mean | |  | Weighted  geometric mean | | |
| --- | --- | --- | --- | --- | --- | --- | --- |
|  |  | SEES | Sensitive |  | SEES | Sensitive | |
| UVI | 1 | 457 | - |  | 250 | - |  |
|  | 2 | 593 | 29.7 |  | 491 | 95.9 |  |
|  | 3 | 728 | 22.9 |  | 683 | 39.2 |  |
|  | 4 | 864 | 18.6 |  | 850 | 24.4 |  |
|  | 5 | 1000 | 15.7 |  | 1000 | 17.6 |  |
| Other gases | 1 | 953 | - |  | 915 | - |  |
|  | 2 | 963 | 1.0 |  | 947 | 3.5 |  |
|  | 3 | 972 | 1.0 |  | 966 | 2.0 |  |
|  | 4 | 981 | 1.0 |  | 980 | 1.4 |  |
|  | 5 | 991 | 1.0 |  | 991 | 1.1 |  |
|  | 6 | 1000 | 0.9 |  | 1000 | 0.9 |  |
| Average sensitive |  |  | 10.2 % |  |  | 20.7 % | |
